# Severe cytokine release syndrome resulting in purpura fulminans despite successful response to nivolumab therapy in a patient with pleomorphic carcinoma of the lung: a case report

**DOI:** 10.1186/s40425-019-0582-4

**Published:** 2019-04-03

**Authors:** Osamu Honjo, Terufumi Kubo, Fumiko Sugaya, Takahiro Nishizaka, Koji Kato, Yoshihiko Hirohashi, Hiroki Takahashi, Toshihiko Torigoe

**Affiliations:** 1 Department of Respiratory Medicine, Sapporo Minami-Sanjo Hospital, Sapporo, Hokkaido 060-0063 Japan; 2 Department of Pathology, Sapporo Medical University, School of Medicine, South 1, West 17, Chuo-ku, Sapporo, Hokkaido 060-8556 Japan; 3 Sakaemachi Dermatology Clinic, Sapporo, Hokkaido 007-0841 Japan; 4 Department of Respiratory Medicine, Teine Keijinkai Hospital, Sapporo, Hokkaido 006-8555 Japan; 5 Department of Respiratory Medicine and Allergology, Sapporo Medical University, School of Medicine, Sapporo, Hokkaido 060-8543 Japan

## Abstract

**Background:**

Immune checkpoint inhibitors (ICIs) have provided more options in the treatment of lung cancer. However, ICIs can cause several unfavorable reactions generally referred to as immune-related adverse effects.

**Case presentation:**

In this report, we present the case of a 52-year-old woman with successful regression of pleomorphic carcinoma of the lung following nivolumab therapy. She developed purpura fulminans (PF) ultimately resulting in amputation of both lower extremities. Blood tests revealed thrombocytopenia with increased serum soluble IL-2 receptor, ferritin, and triglyceride levels suggesting hemophagocytic lymphohistiocytosis (HLH). In addition, serum A disintegrin-like and metalloproteinase with thrombospondin type 1 motifs 13 activity was decreased, suggesting thrombotic thrombocytopenic purpura (TTP). Further detailed analysis revealed severe hypercytokinemia including increased levels of IL-1β, IL-6, IL-10, TNFα, IFNγ, and G-CSF.

**Conclusion:**

The severe systemic inflammatory reaction and impaired peripheral circulation in this patient was attributed to excessive immunological effect induced by nivolumab resulting in cytokine release syndrome (CRS). This is the first report of a patient with multiple pathological conditions including HLH, TTP-like condition, and PF presumably arising from ICI-induced CRS. Further accumulating thoroughly investigated cases would lead to better understanding of the disease and development of reliable cancer immunotherapy.

## Background

Immune checkpoint inhibitors (ICIs) are promising alternatives in cancer treatment in addition to surgery, chemotherapy, and radiotherapy. The basic mechanism of action of ICIs is releasing the brakes of the immune regulation, which is a crucial negative feedback mechanism for avoiding excessive immune reaction. Therefore, ICI therapy has resulted in outstanding success on the one hand and lethal immune-related adverse effects (irAEs) on the other, which are now becoming a major concern in further development of reliable cancer immunotherapy [1]. Hematologic adverse effects in particular are life-threatening but occur relatively infrequently; 7 cases of hemophagocytic lymphohistiocytosis (HLH) following ICI therapy have been reported so far (4 cases of melanoma and 1 case each of urinary bladder carcinoma, Merkel cell carcinoma, and non-small cell lung carcinoma) [2–6]. There has been only 1 reported case of thrombotic thrombocytopenic purpura (TTP) following ICI therapy with ipilimumab [7].

In this report, we present a case of pleomorphic lung carcinoma with significant anti-tumor response to nivolumab therapy. The patient also developed severe cytokine release syndrome (CRS), resulting in HLH and amputation of both lower extremities because of purpura fulminans (PF) probably induced by a TTP-like condition. This is the first report of such a case.

## Case presentation

**Fig. 1:**
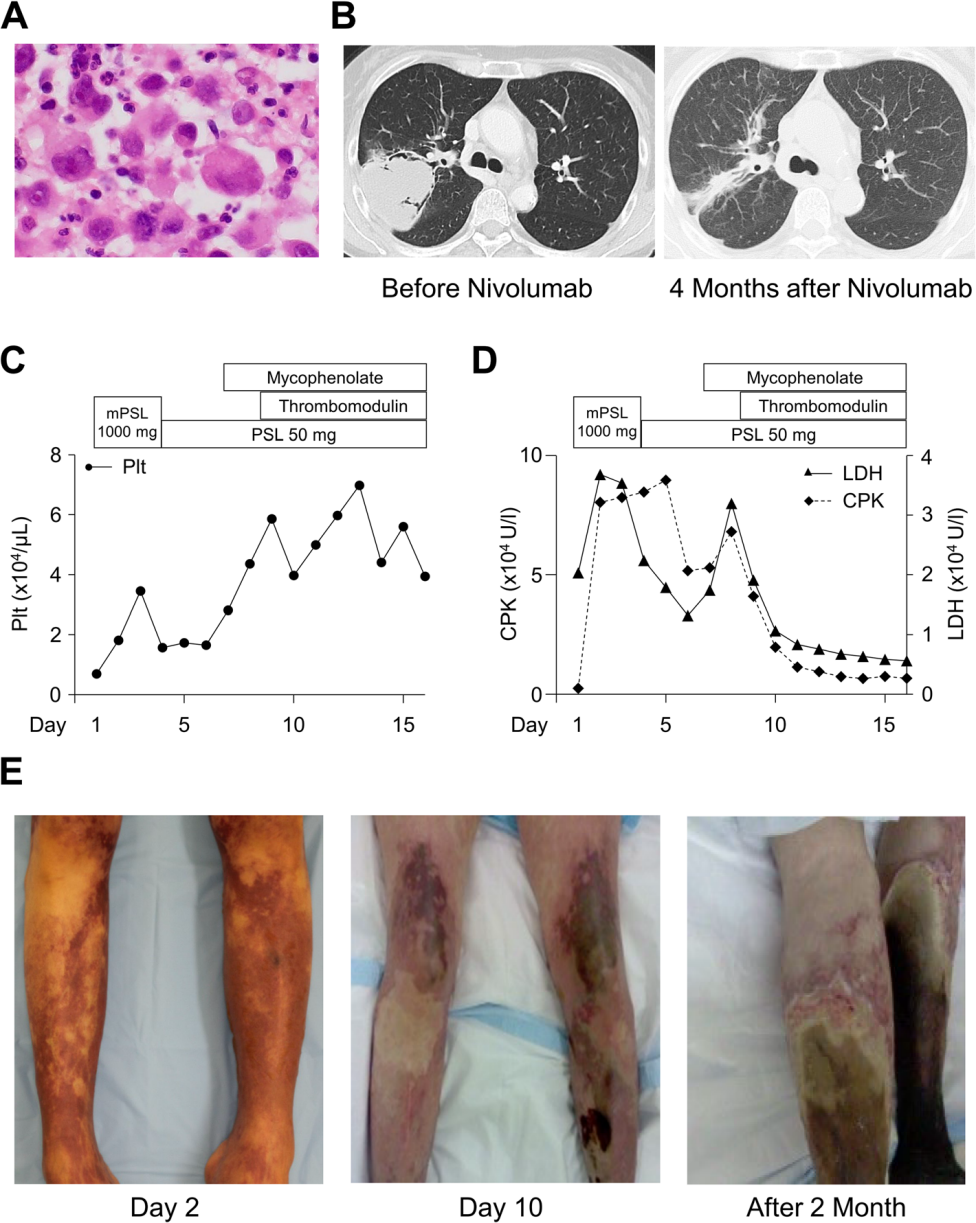
Clinical data and pathological images. **a** Tumor cells with a pleomorphic appearance but are less cohesive. Hematoxylin and eosin staining; original magnification × 200. **b** Computed tomography imaging shows a low-intensity lesion measuring approximately 7 cm in S2 of the right lung (left panel). By 4 months after the last nivolumab administration, no distinctive space-occupying lesion is found (right panel). Graphs representing the trends of **c** platelet count, **d** LDH and CPK from the time of admission (day 1) to day 16. **e** Images of the lower extremities at 2 days (left panel), 10 days (center panel), and 2 months (right panel) after the onset of purpura fulminans

A 52-year-old woman visited a hospital with complaints of right axillary swelling. Computed tomography (CT) imaging identified a space-occupying lesion in the S2 region of the right lung. On histological analysis, the tumor showed high-grade cytological atypia with poor intercellular cohesion (Fig. 1a). Immunohistochemical analysis revealed that the tumor was positive for pan-cytokeratin, vimentin, and thyroid transcription factor 1, but negative for CD45 and CD30, indicating pleomorphic adenocarcinoma of the lung. Programmed cell death ligand 1 was positive in more than 95% of tumor cells (Anti-PD-L1, Clone 22C3; DAKO, Glostrup, Denmark; data not shown). The tumor was evaluated as cT4N2M0 in accordance with TNM classification of Malignant Tumors 8th edition (Union for International Cancer Control, Geneva, Switzerland). The patient underwent four courses of chemotherapy (nab-paclitaxel plus carboplatin). She had fever, polyarthralgia, and muscle soreness. In addition, leukocytosis and thrombocytosis were detected on laboratory testing (data not shown). Based on these clinical features, she was diagnosed as having paraneoplastic syndrome and not an adverse effect of chemotherapy because of paucity of association between symptoms and medication. The patient showed a partial response to chemotherapy based on the Response Evaluation Criteria in Solid Tumors guidelines at which time the tumor was 70 × 55 × 48 mm in size (Fig. 1b; left panel). Then, she was treated with four courses of 120 mg (3 mg/kg) of nivolumab every 2 weeks. Although the patient still had the same symptoms that was diagnosed as paraneoplastic syndrome before, there was no newly developed prodrome or mild noticeable irAEs until the last administration of nivolumab. In addition, laboratory tests performed in each hospital visit did not show any remarkable abnormality.

However, 14 days after the last nivolumab administration, she was admitted to our hospital with complaints of asthenia. The patient felt sick only one day before the admission. She had a fever of 38.6 °C. There was livedo reticularis on the extremities with systemic purpura. Laboratory results on admission are shown in Table 1. The patient had no relevant past medical history or family history including autoimmune disease. In addition, autoantibodies, including antinuclear antibodies, proteinase 3, myeloperoxidase-antineutrophil cytoplasmic, and anticardiolipin antibodies, as well as multiple aminoacyl-tRNA synthetases or anti-CL-β2GP1 antibodies, were not detected. Culture and microscopy of the blood smear did not detect bacterial infection. Conversely, significantly increased serum levels of IL-1β, IL-6, IL-10, TNFα, IFNγ, and G-CSF indicated a state of cytokine storm. Thrombocytopenia and increased serum soluble IL-2 receptor, ferritin, and triglyceride levels would imply possible HLH. Impaired liver function with increased lactate dehydrogenase and creatine phosphokinase together with exacerbated muscle soreness suggested rhabdomyolysis. The activity of A disintegrin-like and metalloproteinase with thrombospondin type 1 motifs 13 (ADAMTS13) was decreased but not less than 10%, which is a criterion for TTP. There was no evidence of infection on blood culture.

**Table 1:** Laboratory test results on admission

Test		Result	Reference Renge (Unit)
Complete blood count	Neutrophils	2630	3500–9700 (/μL)
	Lymphocytes	12.0	18.0–50.0 (%)
	Monocytes	0.0	1.0–8.0 (%)
	Eosinophils	0.0	0.0–7.0 (%)
	Basophils	0.0	0.0–2.0 (%)
	RBCs	492	376–516 × 10^4 (/μL)
	Hemoglobin	12.8	11.2–15.2 (g/dL)
	Hematocrit	39.0	34.3–45.2 (%)
	RBCs MCV	79	80–101 (fL)
	RBCs MCH	26.0	26.4–34.3 (pg)
	RBCs MCHC	32.6	31.3–36.1 (%)
	Platelets	1.1	14.0–37.9 × 10^4 (/μL)
Coagulation	PT	23.3	10.0–13.0 (sec)
	INR	1.94	0.90–1.13
	Fibrinogen	185	170–410 (mg/dL)
	D-dimer	> 60	< 1 (μg/mL)
	TAT	> 120	< 4.0 (ng/mL)
	Lupus anticoagulant	1.3	< 1.3 (normalized ratio)
	vWF activity	> 200	50–150 (%)
	Thrombomodulin	> 32	1.8–3.9 (FU/ml)
Blood Chemistry	Urea	8.9	2.7–7.0 (mg/dL)
	Creatinine	1.81	0.46–0.82 (mg/dL)
	Sodium	137	135–145 (mEq/L)
	Potassium	4.2	3.5–5.0 (mEq/L)
	Chloride	101	98–108 (mEq/L)
	Total protein	5.6	6.5–8.2 (g/dL)
	Albumin	2.8	3.7–5.5 (g/dL)
	Triglyceride	357	50–149 (mg/dL)
	Alkaline phosphatase	878	104–338 (U/L)
	Total bilirubin	4.0	0.3–1.2 (mg/dL)
	Direct bilirubin	3	< 0.4 (mg/dL)
	AST	1556	10–40 (U/L)
	ALT	543	5–45 (U/L)
	LDH	3679	120–245 (U/L)
	CK	8040	5–210 (U/L)
	CK-MB	184.2	< 5.0 (ng/mL)
	Ferritin	3877	21.8–274.7 (ng/mL)
	CRP	41.79	< 0.30 (mg/dL)
Cytokine or bioactive substance	IL-1β	34.9	< 0.928 (pg/mL)
	IL-2	< 15.6	< 15.6 (pg/mL)
	soluble IL-2R	3630	145–519 (U/mL)
	IL-6	1510	< 2.41 (pg/mL)
	IL-10	20.0	< 2.03 (pg/mL)
	IL-12	< 0.78	< 3.15 (pg/mL)
	TNFα	251	< 1.79 (pg/mL)
	INFγ	9260	< 20.6 (pg/mL)
	G-CSF	753	10.5–57.5 (pg/mL)
	ADAMTS-13 activity	37.8	70.0–120.0 (%)
	ADAMTS-13 inhibitor	< 0.5	< 0.5 (BU/mL)

Abbreviations: *MCV* mean corpuscular volume, *MCH* mean corpuscular hemoglobin, *MCHC* mean corpuscular hemoglobin concentration, *PT* prothrombin time, *INR* international normalized ratio, *TAT* turnaround time, *AST* aspartate aminotransferase, *ALT* alanine aminotransferase, *LDH* lactate dehydrogenase, *CK* creatine kinase, *CK-MB* creatine kinase-muscle/brain, *CRP* C-reactive protein

Immediately after admission to the intensive care unit, pulse steroid therapy with methylprednisolone 1000 mg/day for 3 days and systemic prednisolone administration (50 mg/day) were instituted. Thrombomodulin and mycophenolate mofetil was administered thereafter. Any of them could not provide a dramatic amelioration of her general condition, although the laboratory test showed improvement after administration of thrombomodulin and mycophenolate. Trend of platelet count, blood concentration of lactate dehydrogenase and creatine phosphokinase, which were useful as indicator of the clinical state, are depicted in Fig. 1c and d. By 3 days after hospitalization, general hypokinesis of the left ventricular wall resulted in reduced ejection fraction (20%) and aggravation of respiratory distress. Although we did not perform myocardial biopsy, this was probably caused by CRS induced myocarditis, as evidenced by increased levels of the cardiac marker creatine kinase-muscle/brain in the circulation. Continuous hemodiafiltration was also initiated because of renal failure.

By 7 days after hospitalization, the lower extremities were turning black due to circulatory failure (Fig. 1e). Gangrene then spread with infection involving the entire lower extremities, suggesting PF. Amputation of the left and right lower limbs was performed at three and four months after admission, respectively. We could not find active microangiopathy or inflammation in the resected limbs presumably because of the modification by immune suppressants and complete necrosis.

CT evaluation of the pulmonary lesion at 4 months after the last administration of nivolumab demonstrated significant regression; the tumor had become a scar-like lesion (Fig. 1b; right panel) and there was no further progression thereafter. The patient was finally discharged from the hospital at 6 months after admission.

## Discussion

In this case, the patient had pleiomorphic carcinoma treated with programmed cell death-1 (PD-1) inhibitor (nivolumab) and developed severe CRS. Inhibition of PD-1 mediated immunoregulation dramatically enhances anti-tumor immunity in many cancer patients. On the other hand, 30 to 40% of patients who had received nivolumab for non-small cell carcinoma of the lung developed complication of irAEs [8, 9], which often require systemic corticosteroid therapy. It is worth mentioning that patients with irAEs tend to show better progression-free survival in non-small cell lung cancer [10]. This phenomenon suggests that the clinical effects and harmful side-effects of cancer immunotherapy are two sides of the same coin, making cancer immunotherapy that much more difficult. Furthermore, there is no reliable biomarker for estimating clinical efficacy or impending side-effect, although multifarious investigations have been carried out to identify candidate biomarkers from the clinical, pathological, and genetic point of view [8, 9]. For example, paraneoplastic syndrome, a relatively rare condition, is considered to be induced by auto-immune reaction to tumor cells [11]. It would follow, therefore, that this syndrome would be a sign of the immunological effect of ICIs; however, there have been no reports showing any correlation between paraneoplastic syndrome and the efficacy of ICIs. Another candidate predictive marker is morphological atypia, which should be associated with tumor mutation burden, being a recently approved indicator of effect of ICIs. Since pleomorphic carcinoma shows significantly high-grade atypia as found in this case, it might be possible to predict both the beneficial and harmful effects of nivolumab. The problem is that quantifying morphological atypia with high reproducibility is challenging in practice. Currently, routine measurement of serum inflammatory cytokines is not common in daily clinical practice. However, monitoring some types of serum cytokines for a certain period would be helpful in predicting the beneficial or detrimental effects of cancer immunotherapy.

We observed increased serum levels of IL-1β, IL-6, TNFα, IFNγ, and G-CSF, which are known to be pleiotropic inflammatory cytokines that produce a myriad of systemic symptoms. In particular, increased IL-6 is a supposed culprit of myocardial disease and coagulopathy, which was observed in this case, although detailed mechanism is still obscure [12]. Clinically identified fever, thrombocytopenia and increased triglyceride, ferritin, and soluble IL-2 receptor levels suggested the presence of HLH, although the widely used Histiocyte Society HLH-2004 diagnostic criteria were not fully met. However, according to the recently proposed HScore evaluation, a novel sophisticated criterion, the score of this patient was 205 corresponding to more than 90% probability of HLH [13]. IL-6 is thought to be involved in the pathogenesis of HLH [14]. In contrast, an interesting report suggested that highly activated CD8 positive T lymphocytes induced regulatory T cell (Treg) dysfunction in patients with HLH [15]. The mechanism is explained by overconsumption of IL-2 by CD8 positive T cells, resulting in decreased Treg number. Indeed, the number of Tregs is significantly decreased in patients with HLH and recovers to normal levels along with clinical improvement. In line with the focus of this report, the basic mechanism of action of ICIs themselves, activation of CD8 positive cells and suppression of Tregs, can be the fundamental cause of HLH. We suspected the same for secondary TTP as well. We found decreased ADAMTS13 activity, but ADAMTS13 inhibition was not detected. We therefore speculate that the release of multiple inflammatory cytokines hindered the generation of ADAMTS13 from stellate cells, endothelial cells, and platelets, leading to secondary TTP-like microcirculatory impairment resulting in PF, although we cannot exclude a possible subclinical heterogenous ADAMTS13 mutation. This hypothesis is supported by an experimental model where inflammatory cytokines, including TNFα and IFNγ, inhibited synthesis of ADAMTS13 from hepatic stellate cells, a major source of ADAMTS13 [16].

Overall, we propose that all the deleterious hematopoietic adverse effects seen in this case can be attributed to CRS induced by nivolumab-mediated overactivated immune reaction. At the moment, four types of possible mechanisms underlying irAEs have been proposed; overactivation of cellular immune, increasing humoral immune, excessive inflammatory cytokine production and enhancing complement-mediated inflammation [17]. Our current case would be included in “excessive inflammatory cytokine production” pattern. However, we could not identify exact causative factor or specific signaling pathway of this case.

## Conclusion

We encountered a case of multiple pathologies including HLH, TTP-like condition, and PF presumably arising from ICI-induced CRS. To our knowledge, ours is the first report to describe this. CRS has been reported in the setting of autoimmune disease and bacterial or viral infection. However, expanding indications and advancing effectiveness of ICIs would increase the incidence of this disease in cancer patients. Although our current case showed significantly high blood concentration of IL-1β, IL-6, TNFα, and IFNγ when compared with previous reports of CRS [18–20], we did not use the inhibitory agents for inflammatory cytokines in this case because these were not yet recommended in the actual clinical setting at that time. However, a study reported that administration of anti-IL-6 receptor monoclonal antibody (tocilizumab) was useful for treatment of CRS [12]. Apart from tocilizumab, anti-TNFα and anti-IL-1 receptor monoclonal antibodies are also commercially available. Combination of these anti-cytokine medications would be a promising approach for treating CRS. Accumulating a large number of thoroughly examined cases and the resulting better understanding of this disorder should provide safer cancer immunotherapy.

## Abbreviations

ADAMST13: A disintegrin and metalloprotease with thrombospondin type 1 motifs
CRS: Cytokine release syndrome
HLH: Hemophagocytic lymphohistiocytosis
ICI: Immune checkpoint inhibitor
PF: Purpura fulminans
TTP: Thrombotic thrombocytopenic purpura
